# Biological response of extracorporeal shock wave therapy to tendinopathy *in vivo* (review)

**DOI:** 10.3389/fvets.2022.851894

**Published:** 2022-07-22

**Authors:** Yixuan Chen, Kexin Lyu, Jingwei Lu, Li Jiang, Bin Zhu, Xueli Liu, Yujie Li, Xinyue Liu, Longhai Long, Xiaoqiang Wang, Houping Xu, Dingxuan Wang, Sen Li

**Affiliations:** ^1^School of Physical Education, Southwest Medical University, Luzhou, China; ^2^The Affiliated Traditional Chinese Medicine Hospital of Southwest Medical University, Luzhou, China

**Keywords:** extracorporeal shock wave therapy, biological response, tendinopathy, tendon repair, mechanism

## Abstract

Tendinopathy is a degenerative disease of the tendons caused by prolonged overstretching or overuse of the tendons. It accounts for a large proportion of musculoskeletal disorders which can occur in all age groups. The management of tendinopathy is typically conservative. In clinical practice, when other conservative treatments fail, extracorporeal shock wave therapy (ESWT) is normally used as an efficient alternative to surgical management. Several basic studies have shown that ESWT with lower energy flux densities can produce some biological responses *in vivo* to tendinopathy and may accelerate the initiation of the healing process in injured tendons. ESWT has a positive impact on the interactive chain of biological response, enhancing the signaling pathways of angiogenesis through mechanical conduction, and promoting cell proliferation and collagen formation. Finally, it helps tissue regeneration by controlling inflammation. The purpose of this review is to summarize the biological responses generated by ESWT in tendinopathy through a comprehensive review of the published literature. Although ESWT has been used clinically for the treatment of tendinopathies for nearly decades, less is known about the experimental studies of its biological effects on tendon tissue. Further studies on the biological response of ESWT for tendon injuries *in vivo* are needed in the future in order to provide better management to patients.

## Introduction

Tendons are highly differentiated connective tissues that connect muscle to bone in the musculoskeletal system. To effectively transmit muscle forces to the bones, tendons need to withstand the forces generated by muscle loading (1, 2). Hyperextension and overuse of tendons are considered to be primary factors in the development of tendinopathy (3). Tendinopathies account for a great proportion of musculoskeletal disorders and can occur in all age groups. It can cause pain and, in severe cases, even the onset of disability, which can affect the activities of the daily life of humans while also having a huge impact on animals such as dogs, rabbits, rats, etc., especially horses (4–6). Imperfect and slow repair of tendinopathies is the reason for the early retirement of many equines (4).

Depending on the severity of the tendinopathy, its treatment can be divided into two types: surgical and conservative treatment therapy. In general, surgery may be considered for patients who have had poor results with conservative treatment for more than 6 months. Due to the greater complications usually associated with surgical treatment, tendinopathies are most often treated conservatively, which includes local drug injections, medication, exercise therapy, physical therapy, and so on (7). When other conservative treatments for tendinopathy have failed, ESWT is often used as an effective alternative to surgery. ESWT is a mechanical therapy that can alter the chemical environment of the injury site utilizing of mechanical pulsed pressure waves (8). It is capable of reducing and reversing damage to damaged tissues or promoting the healthy growth of normal tissues through mechanical stimulation at the molecular, cellular, or tissue level (9). And it has gradually developed into a way to treat animals in addition to humans (6). In the early 1970 s, ESWT was adopted in medicine as a urinary tract lithotripsy procedure (7). With the rapid development of medical technology, ESWT has gained popularity and acceptance worldwide, so it is gradually being used in the treatment of various musculoskeletal disorders, and in the early 1990 s it was used to treat tendon disorders. In equine medicine, ESWT is used almost exclusively to treat problems related to the musculoskeletal system like tendinopathy (10).

**Figure 1:**
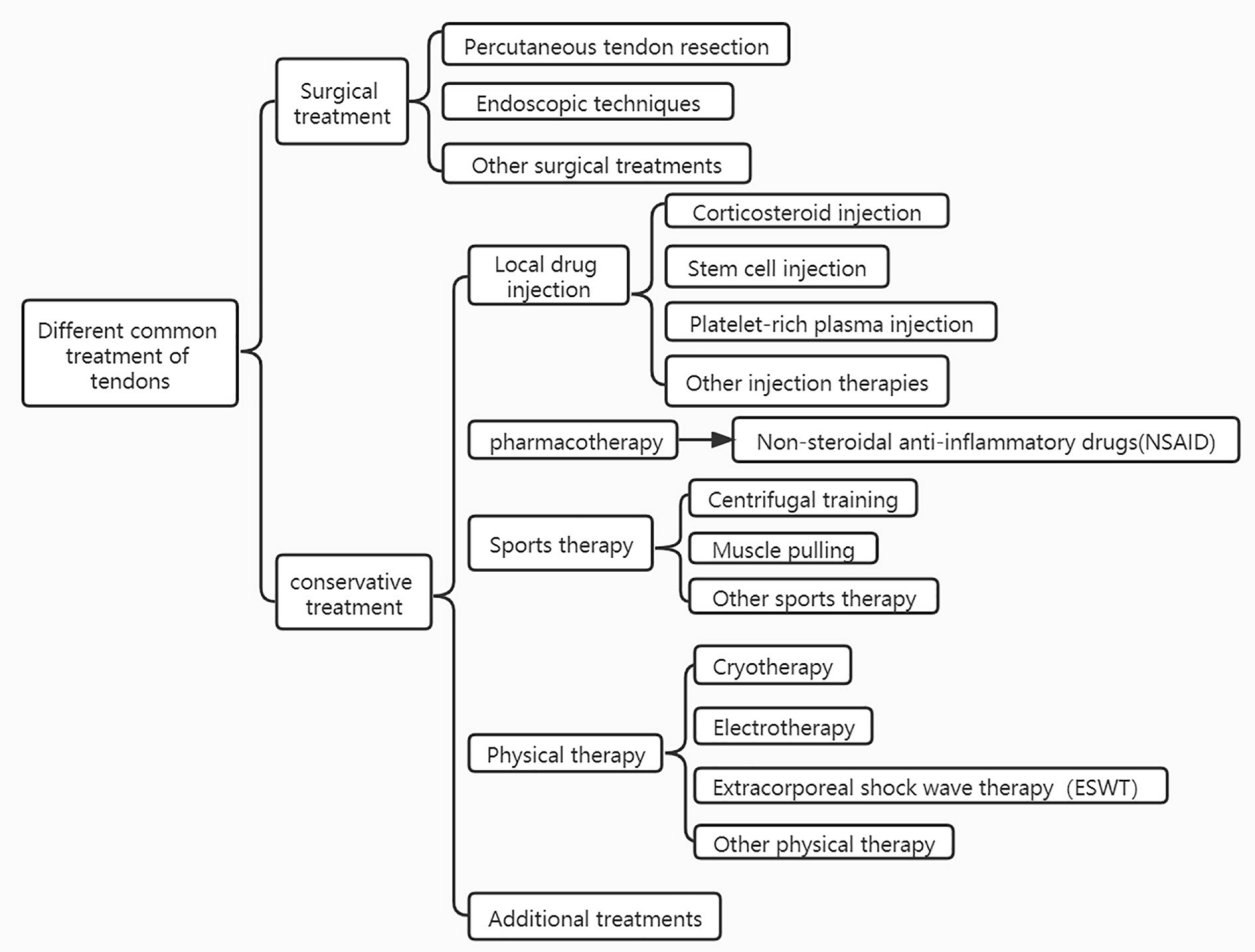
Different treatments for tendon injuries.

Unlike urological treatment, in musculoskeletal therapy, shock waves are not utilized to crush tissue, but to induce extracellular biological responses and tissue regeneration under the microscope. ESWT can facilitate tendon remodeling in tendinopathies by producing mechanical stimulation to eliminate inflammation associated with damaged matrix components and foster catabolic processes Notarnicola and Moretti (2, 11). Nonetheless, the effectiveness of ESWT may depend on the stage of the tendinopathy, and it seems to be more appropriate for the later stages of tendon degeneration (12). Different frequencies of shock waves have different efficacy in dealing with tendinopathies. It was shown that lower frequencies were more effective in healing tendinopathy, but higher frequencies were not. The advantage of low-frequency shock waves is that they are safer and more effective, so they are mostly used to treat tendinopathies. An animal study showed that high-energy shock waves may cause potential damage to tendon tissue, such as inducing fibrinoid necrosis, paratendinous fibrosis, and inflammatory cell infiltration in normal tendons (13). Therefore, it is recommended that shock waves with an energy flux density >0.28 mJ/mm2 are not used clinically for the treatment of tendinopathies. ESWT can be divided into 2 types, radial and focused. Radial shock waves have a more superficial effect, and focused shock waves reach their maximum energy at a focal point located deep in the body tissue. To date, most studies on ESWT for tendinopathy have been performed with focused shock waves (14). With the advantages of non-invasiveness, relatively low cost, and low incidence of complications, shockwave therapy may be an effective alternative to other conservative and surgical treatments for patients with chronic wounds (15). The success rate of shockwave therapy for tendinopathy ranges from 65 to 91% (16). Until now, many successful applications have been reported in the literature. As evidence continues to accumulate, more animal patients may benefit from ESWT (17). Despite the encouraging progress that has been made, the biological responses generated by ESWT in tendon disease remain to be fully understood. Different treatments for tendon injuries are shown in Figure 1.

This review summarizes the *in vivo* biological response to ESWT in the three phases of tendon repair through a comprehensive review of the published literature on the subject, with a view to providing evidence to support the future use of ESWT in tendinopathies and to address, to some extent, the controversial issues surrounding its application.

### Search strategy

**Figure 2:**
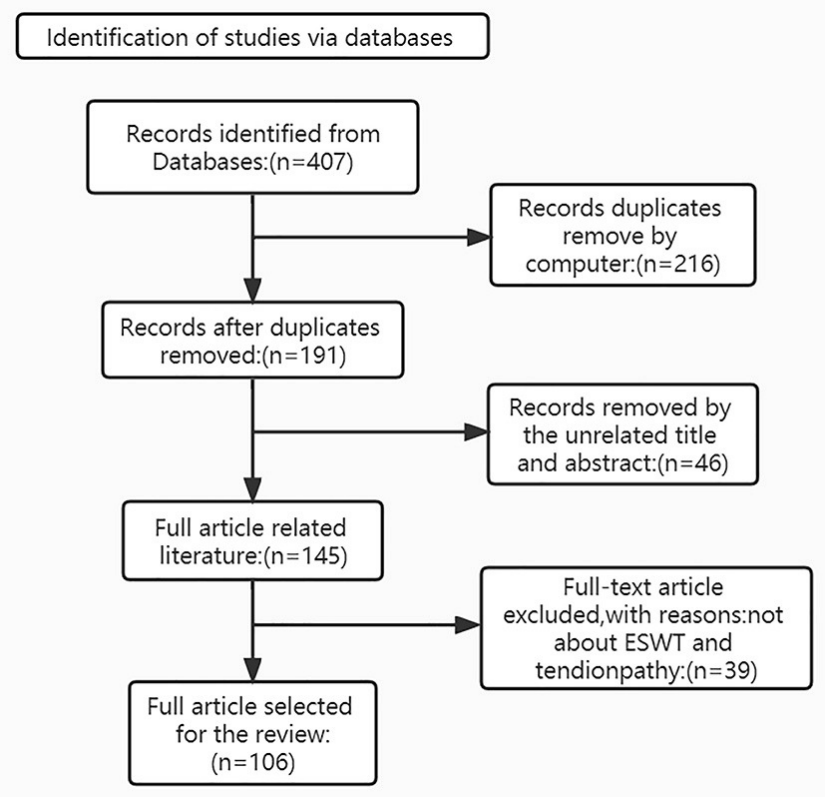
Article Retrieval Flow Chart with inclusion and exclusion process.

(i) Search site: Articles are from PubMed, a database of papers on biomedical science. (ii) Database: MEDLINE. (iii) Keywords: tendon injury; tendinopathy; extracorporeal shock wave therapy (iv) Boolean algorithm: (Extracorporeal shock wave therapy) AND (Tendon injuries OR Tendinopathy OR Tendon repair). (v) Retrieval timeframe: We searched the selected journals published from 1979 to 2022. (vi) Inclusion and exclusion criteria: Articles were included if the topic is related to extracorporeal shockwave therapy and tendon repair, and the article type was a review or an experimental paper. The search process is shown in Figure 2.

## Comparison of the clinical efficacy of ESWT in different common tendinopathies

With the advancement of technology, in orthopedics, ESWT is being used relatively rapidly in the treatment of many tendinopathies, such as plantar fasciitis (PF), lateral epicondylitis (LE), calcific tendinitis (CT), and patellar tendinopathy (PT) and so on. The vast majority of applications for ESWT in the treatment of tendinopathies have shown positive clinical efficacy *in vivo*, ranging from 65 to 91% success rates (16, 18).

PF is a common cause of heel pain. Among the several conservative treatment options for PF, ESWT is considered the gold standard. And PF also happens to be one of the indications with the best results after applying ESWT, along with insertional tendinopathy of the Achilles tendon and mid-portion Achillodynia (19, 20). We can learn in a meta-analysis that Fan et al. found that moderate energy ESWT (0.12–0.25 mJ/mm2) had similar effects to low energy ESWT (0.06–0.11 mJ/mm2) in improving pain scores in Achilles tendinopathy, suggesting that further studies are needed to determine the optimal energy level for treating Achilles tendinopathy. However, ESWT still offers significant advantages over other non-surgical treatments in reducing pain and improving function in patients with Achilles tendinopath (21). Meanwhile, plantar fasciotomy and ESWT have shown similar positive functional effects for the treatment of PF, and yet, ESWT did not incur surgical risks including surgical pain (14). In a randomized double-blind trial, Rompe et al. found that low-energy focused ESWT with a total of 3,000 shocks and an EFD of 0.08 mJ/mm2 demonstrated effective efficacy in PF, significantly reducing pain and improving function (22). Lai et al. used focused ESWT with an EFD of 0.29 mJ/mm2 held for 25 min in their experiment, reaching a total of 1,500 shocks in each treatment, and in the final results the ESWT showed better efficacy than corticosteroid injections (23). Çaglar Okur et al. used radial ESWT in a randomized controlled study, and after delivering 400 pulses at 12-Hz and 2.0-bar pressure to the painful spot, PF showed remarkable improvement in walking, morning and night pain, and maintenance of foot function and health (24). In addition to the above examples, there are many published papers that report the positive effects of ESWT on PF. For instance, the protocol was found to be effective in studies by Gleitz and Hornig (25–28). On the other hand, a few reports such as the studies of Buchbinder et al. suggested the opposite conclusion. In their research, ESWT showed a non-positive effect on PF (29–31). Divergence in the efficacy of ESWT for PT across studies may be related to many factors, including differences in subject populations, heterogeneity of treatment parameters, and different machine designs, among others. In the future, we need further randomized trials to improve the evidence in certain areas.

Compared to PF, LE has a lower success rate and is the least effective of all recognized indications (32). Nevertheless, some studies still show positive results, for example, in a randomized controlled trial, where Rompe et al. concluded that ESWT improved upper extremity function and clearly reduced pain during resistant wrist extension in patients with LE. In their study, they used focused low-energy ESWT with an EFD of 0.09 mJ/mm2 and a repetition frequency of 4 Hz to treat LE. In the treatment group, 65% of patients had at least 50% pain reduction, while only 28% of patients in the sham group achieved pain reduction (33). At the same time, there is evidence of a negative effect of ESWT on the efficacy of LE. A randomized multicenter trial showed that the overall success rate of ESWT for LE at 3 months was 25.8, or 31.7% when measured by the Rawls and Maudsley score. Similar success rates were achieved in the placebo group, with 25.4 and 33.1%, respectively. This suggests that there was little positive impact on the success rate of ESWT for LE (34). And in a prospective randomized study comparing steroid injections with ESWT, we can learn that steroid injections improved symptoms more than ESWT at 3 months after the end of treatment (35).

In ESWT applications for CT, a randomized controlled trial by Gerdesmeyer et al. showed that high-energy (0.32 mJ/mm2) ESWT could produce better results than low-energy ESWT, although low-energy ESWT could also reduce calcified deposits. In this study, the high-energy group was given 1,500 shock waves of 0.32 mJ/mm2 per session, whilst the low-energy group took 6,000 shock waves of 0.08 mJ/mm2. We know from the final results of this study that both high-energy and low-energy ESWT had beneficial effects on shoulder function and reduced calcification size, but that high-energy ESWT was superior to low-energy ESWT for CT patients (36). There has been a paper comparing different approaches to the application of ESWT (radial vs. focused) for patients with CT. Cacchio et al. performed a randomized controlled trial of radial and focused ESWT and showed that radial ESWT was more efficacious for CT (37).

Similar to CT or other tendinopathies, ESWT is not a first-line treatment for PT, but it is a safe and promising treatment for them (38, 39). In the case of ESWT for PT, satisfactory results were obtained in 73.5% of cases (11). In the first randomized controlled trial to comparing the efficacy of FSWT and RSWT for PT, van der Worp et al. compared 21 patients treated at 2,000 sessions of 0.12 mJ/mm2 focused ESWT with 22 patients treated at 2,000 sessions of 2.4 bar radial ESWT, using similar intensities in both groups. The results showed no difference in the efficacy of focused ESWT and radial ESWT for PT (40). Conflicting evidence exists regarding the clinical effectiveness of ESWT in the treatment of PT. Evidence from some studies suggests that ESWT is effective in treating PT, while in other clinical studies, ESWT showed little to no improvement (41, 42). For instance, a retrospective study showed a moderate level of evidence suggesting the efficacy of ESWT and placebo ESWT was equal in terms of pain suppression and restoration of function at short and intermediate (5–6 months) follow-up. In contrast, a low level of evidence suggests that ESWT is superior to control conservative treatment in terms of functional and pain outcomes at long-term follow-up (2–3 years) (43). The reasons for the contradiction may be: the lack of objective diagnostic criteria for PT; the fact that ESWT may be effective only for certain stages of tendinopathy; and the lack of uniform treatment parameters for ESWT (41). Hence, further studies are still needed in the future to develop objective diagnostic criteria for PT and standard treatment parameters to ensure that satisfactory treatment outcomes are achieved.

In addition to the tendinopathies we mentioned above, ESWT has been used in the treatment of other tendinopathies with an average success rate of between 60 and 80% (44). Based on the inspiring results of clinical and experimental studies, the potential of ESWT is becoming apparent. There is an increasing interest in the mechanism of action of using ESWT in the treatment of tendinopathies. In the future, we should make improvements in accordance with evidence-based medicine and make more efforts in order to further demonstrate the effectiveness and safety of shock wave therapy in the treatment of tendinopathies. Comparison of the clinical efficacy of ESWT in different common tendinopathies is shown in Table 1.

**Table 1:** Comparison of the clinical efficacy of ESWT in different common tendinopathies.

**Tendons in different parts**	**Therapeutic effect**
Plantar fasciitis (PF)	PF is one of the indications with the best results after application of ESWT. Among several conservative treatment options for PF, ESWT is considered the standard of care, resulting in lower pain and better functional outcomes for AT patients. Meanwhile, plantar fasciotomy and ESWT have shown considerable functional effects for the treatment of PF.
Lateral epicondylitis (LE)	Among the indications for ESWT in the treatment of tendinopathy, LE has the worst efficacy.
Calcific tendinitis (CT)	ESWT is as effective as or better than surgery in the treatment of CT. Radial ESWT is considered to be more effective in the treatment of CT than focused ESWT. On the other hand, high-dose ESWT is significantly more effective than low-dose ESWT in the treatment of CT, but low-energy ESWT can also dissolve calcium deposits.
Patellar tendinopathy (PT)	ESWT is not the first line of treatment for PT, but for them ESWT is also a safe and promising treatment with positive effects on pain and function.
Achilles tendinopathy (AT)	Insertional AT and mid-portion Achillodynia are also among the indications with the best results after the application of ESWT. Patients who received ESWT for AT had significantly better pain and functional outcomes than those who received other treatments, including sham ESWT, eccentric training, and other traditional treatments.

## Biological responses caused by ESWT in the three phases of tendon repair

Tendinopathy has a complex pathophysiological mechanism, which can cause pain and, in severe cases, even disability. With the advancement of medical technology, ESWT is increasingly used to treat tendinopathies. So far, the biological basis of research on ESWT has focused on pain relief, disruption of calcification, and tissue regeneration (12). First, some studies have shown that ESWT has analgesic effects similar to those of capsaicin (45). In the studies by Dahlberg, Imboden, and MacKay et al. the application of ESWT improved pain well in equine patients, and this improvement could be explained by a reduction in lameness or changes in imaging evidence in equines (6, 46, 47). Meanwhile, more studies have pointed out several possible mechanisms by which ESWT relieves pain: ESWT may produce analgesic effects by inhibiting the activity of the serotonergic system; inducing selective innervation of peripheral nerves, which is involved in pain modulation; or it may relieve pain through hyperstimulation, which is the most frequently applied theory (48–50). In the study using low-energy ESWT for calcific tendinopathy, by affecting the patient's pain as an objective, the experience gained by the researchers was consistent with the main points of Melzack's theory of overstimulation analgesia theory: brief stimulation relieves chronic pain in the long term; moderate to strong stimulation relieves pre-existing pain; and this stimulation must act directly on the point of pain. The hyperstimulation produced after the application of ESWT is a factor that can activate sensory input to specific nerves or tissues in small fibers. When this stimulation is applied to the treatment site, it can bring more input to the central biasing mechanism. This closes the gate to the input from the selected body site, weakening the signal transmitted to the brainstem and ultimately affecting the transmission of pain (51, 52). Secondly, ESWT is also able to disrupt calcification in the tendons in a manner comparable to urolithotripsy. Some *in vivo* studies have shown that calcification in shoulder tendinopathy is disintegrated after ESWT (12). Most importantly, several basic studies have shown that ESWT with lower energy-flux density can aid in tissue regeneration and thus accelerate the initiation of the injured tendon healing process (53).

In the experiments by Waugh et al. they used microdialysis to investigate the real-time biological response of pathological and healthy tendons to ESWT, giving us insight into the biological mechanisms that support the clinical effects of ESWT for humans *in vivo*. Among the articles we consulted, this is one of the earliest studies documenting the direct *in vivo* biological response of pathological and healthy tendons to ESWT. The findings demonstrate that the mechanical stimulation offered by ESWT may contribute to the initiation of regeneration of tendons in tendinopathies, as it promotes inflammatory and catabolic processes related to the removal of injured matrix components (54). Similarly, the effectiveness of ESWT *in vivo* was also proved in a study by Heimes et al. They treated fertilized eggs with 0.12 mJ/mm2 and a total of 500 pulses of ESWT in the chorioallantoic membrane (CAM) assay and thus performed an *in vivo* analysis of angiogenesis and the immune response of the biomaterials, confirming the beneficial effect of ESWT on angiogenesis while showing a reduction in inflammation (55).

**Figure 3:**
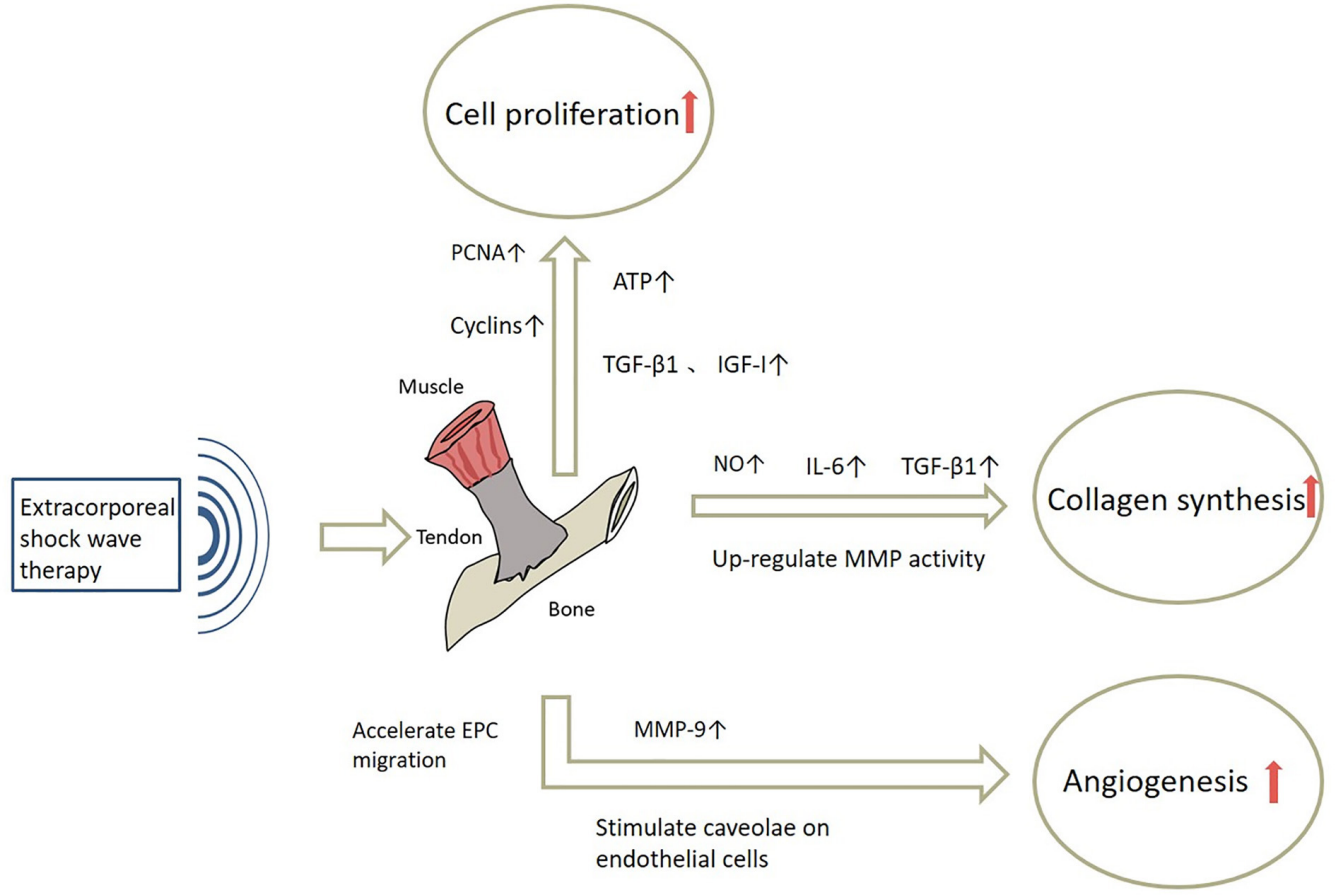
The mechanisms of ESWT in tendinopathy. NO, Nitric Oxide;IL-6, Interleukin-6; MMP, Matrix metalloproteinase; PCNA, Proliferating Cell Nuclear Antigen; ATP, Adenosine triphosphate; MMP-9, Matrix metallopeptidase 9.

**Figure 4:**
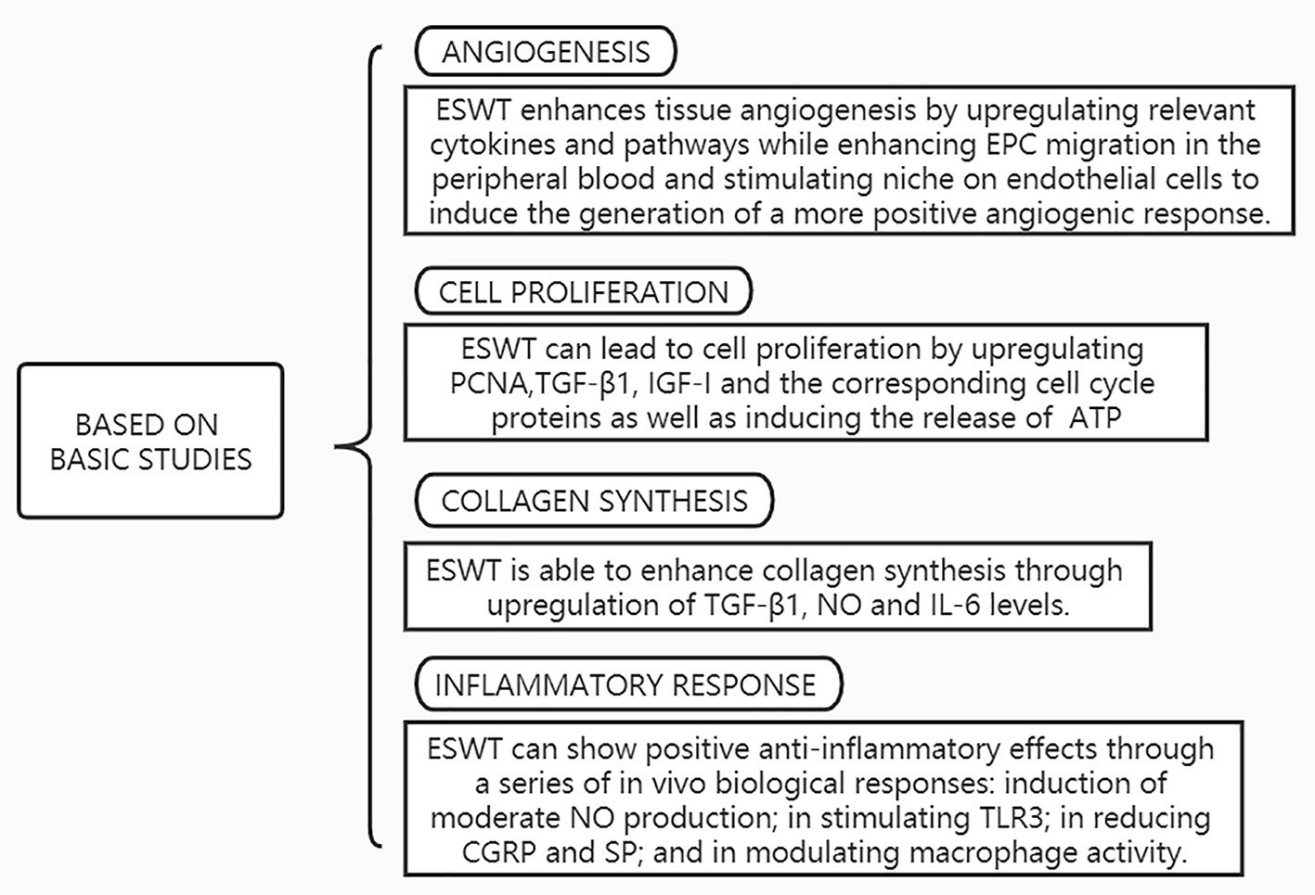
Effects of ESWT in tendinopathy according to basic studies. EPC, Endothelial progenitor cells; TGF-β, Transforming growth factor-beta; IGF-I, Insulin-like growth factor-I; PCNA, Proliferating Cell Nuclear Antigen; ATP, Adenosine triphosphate; NO, Nitric Oxide;IL-6, Interleukin-6; TLR3, Toll-Like Receptor 3; CGRP, Calcitonin gene-related peptide; SP, Substance P.

The process of tendon healing is not straightforward. It is generally divided into three stages: inflammation, cell proliferation and remodeling (3). In the earliest stages of inflammation, inflammatory cells enter the site of injury, blood vessel permeability is increased, and then angiogenesis begins. During the proliferative phase, tendon cells are stimulated and tendon cells gradually migrate into the wound while proliferating and begin to accelerate the process of collagen synthesis. Ultimately, remission of the inflammatory response can facilitate extracellular matrix proteins (ECM) remodeling and promote the regression of tendinopathy during the remodeling phase (56). The mechanisms of ESWT in the treatment of tendinopathy are shown in Figure 3 and effects of ESWT in tendinopathy according to basic studies are shown in Figure 4. Simultaneously, the biological responses embodied by ESWT in different animal experiments are shown in Table 2.

**Table 2:** Summary of the biological responses embodied by ESWT in diverse animal experiments.

**References**	**Animal type**	**Models establish**	**Dosage**	**Time post operation**	**Outcome**	**Conclusion**
Bosch et al. (57)	Shetland ponies	Flexor-extensor tendons and suspensory ligaments	EFD of 0.14 mJ/mm2 for 600 impulses	3 h, 6 weeks	MMP3↓MMP13↑ (3 h later)COL1↑MMP13↑MMP14↑ (After 6 weeks)	The disorder of collagen matrix and the increase of COL1 gene expression may explain the effectiveness of ESWT in chronic tendinopathy
Waugh et al. (2)	*In vitro*	Middle Achilles tendon and patella	2,500 impulses administered at 8 Hz. The total energy delivered was 160 mJ/mm.	1, 2, 3, 4 h	IL-6↑IL-8↑MMP-2↑MMP-9↑	The mechanical stimulation provided by ESWT may help tendon remodeling in tenopathy by promoting inflammatory and catabolic processes associated with the removal of damaged matrix components.
Aschermann et al. (58)	*In vitro*	Human foreskin for routine prepuce surgery	EFD of 0.136 mJ/mm2 for 0, 375, 750, and 1,500 impulses, respectively	24 h, 48 h	Motility of HaCat↑Number of fibroblasts↑EC↑	Shock waves induced a biological cascade of complex cellular events that efficiently induced wound healing processes in the respective cellular models.
Chen et al. (59)	Sprague-Dawley rats	Achilles tenotomy	EFD of 0.16 mJ/mm2 for 0, 200, 500, and 1,000 impulses, respectively	1, 4, 6, 8, 12 weeks	PCNA↑TGF-β1↑IGF-I↑Vascular numbers ↑cell proliferation↑	ESWT with 200 pulses can promote the healing of collagenase-induced tendinitis, while ESWT with more than 200 pulses can inhibit tendon repair.
Vetrano et al. (18)	*In vitro*	ACL excision and reconstruction with semitendinosus tendon graft	EFD of 0.14, mJ/mm2 for 1,000 impulses	1, 4, 8, 12 days	Collagen synthesis↑Cell proliferation activity↑Stiffness ↑	Shock wave treatment promoted the cell growth and collagen synthesis of primary cultured human tendon cells
Uzun et al. (60)	New Zealand female rabbits	Achilles tenotomy	ESWT (3 doses/28 days, 1st dose: 0.12 mJ/mm2, 15 Hz, 300 impulses; 2nd dose: 0.14 mJ/mm2, 15 Hz, 500 impulses; 3rd dose: 0.14 mJ/mm2, 15 Hz, 500 impulses)	28 days	Cell proliferation↑Collagen synthesis↑Deformability↑	ESWT and PEMF have the same effect on the quality of Achilles tendon and increase cell proliferation in the wound area. ESWT is effective in the early recovery phase, that is, the proliferation phase.
Orhan et al. (61)	Wistar albino rats	Achilles tenotomy	500 shocks in 5 min with an intensity of 15 kV.	4, 12 weeks	Collagen synthesis↑Vascular numbers ↑Cell proliferation activity↑Tensile Strength↑	Anti-angiogenic treatment during early tendon healing is beneficial for tendon quality following injury
Wang et al. (62)	Mongrel dogs	Achilles tenotomy	EFD of 0.18 mJ/mm2 for 1,000 impulses	4, 8 weeks	Vascular numbers ↑Miscellaneous fibers↓	Low-energy shock wave enhanced the phenomenon of neovascularization at the bone-tendon junction in dogs.
Hatanaka et al. (63)	*In vitro*	Cultured HUVECs	treated with 800 shots of low-energy SW (1 Hz at an energy level of 0.03 mJ/mm2)	24, 48 h	VEGF↑eNOS↑Erk1/2↑AktCaveolin-1↑β1-Integrin↑	ESWT enhances angiogenic signaling pathways by stimulating small concavities on the endothelial cell membrane and subsequently activating local adhesion pathways.
Wang et al. (64)	New Zealand white rabbits	Biopsy of the Achilles tendon bone unit	EFD of 0.12 mJ/mm2 for 500 impulses	24 h, 1, 4, 8 weeks	Vascular numbers ↑eNOS↑VEGF, ↑PCNA↑	ESWT produced significantly more neointimal and angiogenesis-related markers, including eNOS, VEGF, and PCNA.
Mariotto et al. (65)	Dogs	Tendons of dogs	EFD of 0.03 mJ/mm2 for 500 impulses; EFD of 0.1 mJ/mm2 for 500 impulses	4, 8 weeks	NO↑eNOS↑NF-κB↓	The blood flow around the treated area immediately increases.
Weihs et al. (66)	Male Sprague-Dawley rats	C3H10T1/2 murine mesenchymal progenitor cells, primary human adipose tissue-derived stem cells, and a human Jurkat T cell line	*In vitro*, 10 −300 pulses of shock waves using energy flux densities between 0.03 and 0.19 mJ/mm2 at 3 Hz were applied.	1, 5, 10 days	Release of ATP↑Erk1/2↑cell proliferation↑	ESWT triggers the release of cellular ATP, which subsequently activates purinergic receptors and ultimately enhances proliferation *in vitro* and *in vivo via* downstream Erk1/2 signaling.
Chao et al. (67)	Sprague-Dawley rats	Achilles tenotomy	0.36 mJ/mm 2 with 50 and 100 impulses	6, 24, 48, 96 h and 7 days	PCNA↑(at 6 and 24 h) NO ↑(at 24 h) collagen type I ↑collagen type III↑ and TGF-β1↑(at 24 h) collagen synthesis↑(at the 7th day)	Related tenocyte proliferation is mediated by the early up-regulation of PCNA. The synthesis mechanism of collagen and mRNA levels may be mediated by the release of endogenous NO and the up-regulation of TGF-β1.

*↑, significant increase;↓, significant decrease; ESWT, Extracorporeal shock wave therapy; SW, shock wave; EFD, Energy Flux Density; MMP, Matrix Metalloproteinase; TGF-β1, Transforming Growth Factor-β1; COL1, Type I Collagen; IL, Interleukin; PCNA, Proliferating Cell Nuclear Antigen; IGF-I, Insulin-Like Growth Factor I; VEGF, Vascular Endothelial Growth Factor;Erk1/2, Extracellular Regulated Protein Kinases1/2;ACL, Anterior Cruciate Ligament; NO, Nitric Oxide; NF-Kb, Nuclear Factor Kappa-B; ATP, Adenosine Triphosphate*.

### ESWT increases angiogenesis in the inflammatory phase

The angiogenic response is a key event in the biology of tendon healing (15). A pilot study has shown that shock waves can improve blood flow and perfusion during the inflammatory phase of tendon repair, accelerating angiogenesis *in vivo* without any adverse effects (68, 69). The neovascularization phenomenon was enhanced by ESWT in the dog experiments of Wang et al. They found new capillaries in all study samples obtained at week 4 and week 8 after shockwave application, respectively. The size and shape of the new capillaries observed were similar on both occasions. It suggests that most of these new vessels may persist and proliferate for up to 8 weeks after shockwave application (62). And Kersh et al. have also shown that ESWT significantly increases angiogenesis in the superficial digital flexor tendon (SDFT) of horses. SDFT injuries are common in most equines. The quality of healing of the SDFT determines whether the equine will recover to near normal athletic levels. Their study found significantly more capillaries in the ESWT-treated tendons than in the untreated controls (70). These increases in new blood vessel formation all indicate that ESWT can induce a beneficial tendon healing response. In our summarized article, ESWT can promote angiogenesis by mobilizing endogenous circulating endothelial cells that are eligible for angiogenesis and is also able to aid angiogenesis by stimulating caveolae on endothelial cells and upregulating relevant cytokines and signaling pathways.

Initially, ESWT promotes angiogenesis by enhancing the migration of endothelial progenitor cells (EPC) in peripheral blood through three pathways. First, ESWT accelerates EPC migration and angiogenesis by increasing the concentration of hypoxia-inducible factor 1α (HIF-1α) and thus upregulating stromal cell-derived factor-1 (SDF-1) and vascular endothelial growth factor (VEGF).SDF-1 is a major chelator that recruits and major chelator attributed to EPCs, which enhances EPC migration. VEGF is the most potent endothelial-specific mitogen that promotes angiogenesis at the molecular level and also contributes to the active mobilization of EPCs (71, 72). Second, two cytokines, SDF-1 and VEGF, are able to be directly affected by ESWT, obtaining an upregulation of concentration, which then enhances the migration of EPCs and ultimately increases angiogenesis (72). Third, ESWT may also lead to EPC migration through activation of the mechanosensing complex. The mechanosensing complex consists of VEGFR-2, VE-cadherin, and pecam1, and its activation significantly enhances the PI3K-Akt-eNOS and extracellular signal-regulated protein kinases 1 and 2 (ERK1/2) signaling pathways, which in turn leads to EPC migration and therefore enhances EPC survival and angiogenesis (73).

Then, ESWT is able to induce the generation of angiogenic responses by stimulating caveolae on endothelial cells. After acting on tendons, ESWT stimulates caveolae on endothelial cells, which then induces phosphorylation of Caveolin-1 and activation of β1-Integrin, two mechanotransduction proteins, thereby promoting phosphorylation of its downstream pathways Ekr1/2 and Akt, while positively upregulating concentrations of VEGF and endothelial nitric oxide synthase (eNOS), and finally enhancing the signaling pathway of angiogenesis (63).

Additionally, neovascularization as an invasive process requires the proteolytic activity provided by matrix metalloproteinase (MMP) to degrade the ECM and thus aid in the sprouting of neovascularization. Matrix metallopeptidase 9 (MMP-9) is an angiogenic, endothelial cell migration stimulating factor, and in addition, it can activate some cytokines, such as VEGF. These can all aid in the angiogenic response. It has been shown that MMP-9 expression is increased after ESWT. Therefore, ESWT is thought to enhance tissue angiogenesis by increasing the level of MMP-9 (55). In short, ESWT accelerates angiogenesis, thus promoting an increase in nutrients and further accelerating tendon healing.

### ESWT stimulates tendon cell proliferation and increases collagen synthesis in the cell proliferation phase

Cell proliferation is an extremely critical biological process in the process of tendon healing by ESWT, which can initiate a cell regeneration cascade by inducing mitosis and proliferation of fibroblasts, ultimately producing a positive healing outcome (58). In our review of the literature, ESWT can lead to cell proliferation by upregulating proliferating cell nuclear antigen (PCNA), transforming growth factor-beta 1 (TGF-β1), insulin-like growth factor-I (IGF-I) and the corresponding cell cycle proteins as well as inducing the release of adenosine triphosphate (ATP).

Above all, ESWT is able to proliferate cells through the upregulation of early PCNA, which was originally thought to be a nuclear antigen in systemic lupus erythematosus (SLE) patients with autoimmune diseases. The expression of its gene was associated with the proliferation of rat tendon fibroblasts (67, 74). After ESWT, gene expression of PCNA is significantly upregulated, which activates the signal transduction and activation of transcription factor-3 (STAT3) signaling pathway (59). STAT3 is a key mediator of intracellular signaling that induces transcriptional activation of various growth-promoting genes. Finally, the activation of STAT3 signaling pathway leads to cell proliferation (75).

Furthermore, TGF-β1 and IGF-I have a crucial role in the promotion of tendon cell proliferation and tissue regeneration by ESWT. Tendon tissue can assist tendon repair by converting mechanical stimulation of ESWT into biochemical signals *via* TGF-β1 and IGF-I. Meanwhile, among the growth factors that regulate tendon repair, TGF-β1 and IGF-I have been found to have the function of proliferating tendon cells, while regulating collagen metabolism, and then promoting tendon regeneration (59).

Moreover, some studies have indicated that during the process of tissue healing, structural recovery is closely related to both mitotic and migratory cellular behavior, and mitosis is one of the ways in which cells of an organism proliferate. According to some basic experiments, ESWT is able to promote cell proliferation by upregulating the corresponding cell cycle proteins, inducing cell cycle entry into the S phase and G2/M, and reducing G0/G1 phase. Expression of Cyclin-dependent kinases1 (CDK1) and CyclinA2 (CCNA2) can aid the healing process, and the positive effect of S-phase progression on the healing process in eukaryotic cells could support this idea.CDK1 is a key regulator of the G1/S and G2/M transitions of the eukaryotic cell cycle, while CyclinA2 is a regulator of CDK. ESWT can increase CDK1 expression by upregulating CCNA2, resulting in the promotion of cell cycle G1/S and G2/M transitions. On the other hand, CyclinB1 (CCNB1) and CyclinB2 (CCNB1), two cell cycle proteins important for the maintenance of mitotic arrest, were also significantly increased after ESWT treatment. Finally, at the end of ESWT application, the earliest initiation of mitotic machinery at 24 h after application and enhanced cell proliferation after 72 h were presented (58).

As a final note, shock waves can enhance ERK1/2 signaling by inducing the release of ATP, thereby accelerating cell proliferation. Although we do not know the mechanism of ATP release from shockwave-treated cells, shockwave treatment does trigger the release of cellular ATP. ESWT induces the release of ATP that binds to purinergic receptors, which in turn leads to a downstream signaling event, and thus Erk1/2 signaling is enhanced, ultimately leading to cell proliferation (66).

Although tendon cells are the major cellular component of tendons, 95% of the normal tissue of tendons is formed by ECM, of which collagen is the major component (18, 76). In the experimental rat model established by Orhan et al. the rat Achilles tendon showed an increase in not only fibroblast activity but also collagen synthesis with the application of ESWT (77). As a major component of the fibrous system, collagen synthesis and deposition is important for tendon healing. It has high tensile strength and stiffness and is therefore very critical in later events of healing, such as tendon consolidation after injury (18). The positive effect of ESWT on tendon collagen synthesis was also shown in a study by Hsu et al. They treated a rabbit patellar tendon model with ESWT and found more collagen synthesis in the ESWT group by comparing the results of hydroxyproline concentrations (78). All of these are suggesting that ESWT-treated tendons undergo positive changes at the cellular level. During the cell proliferation phase, ESWT can facilitate collagen synthesis by upregulating or activating relevant cytokines. Several research groups have demonstrated that in many cell types, TGF-β1 increases collagen production and secretion while stimulating the expression of ECM (67, 79–81). And ESWT is able to induce TGF-β1 production through mechanical stimulation (67, 82). Hence, ESWT can increase collagen synthesis in tendon cells by upregulating TGF-β1.

Thereafter, ESWT can increase collagen synthesis by up-regulating Nitric oxide (NO) concentration. Low-intensity short shock wave stimulation causes tendon fibroblasts to produce high levels of NO immediately within 24 h (67). Although the mechanism by which NO affects collagen synthesis remains unclear, this mechanism may be related to TGF-β1. NO can lead to the activation of latent TGF-β1 (83). This increases collagen production. NO can also induce other cytokines such as basic fibroblast growth factor (b-FGF), nuclear transcription factorκB (NF-κB), and insulin-like growth factor (IGF), which may play a significant role in collagen synthesis (84–87). In contrast, longer shock wave stimulation did not result in significant changes in NO levels. On the other hand, ESWT may activate TGF-β1 latent in the ECM by upregulating MMP activity, which then results in an increase in collagen synthesis (88).

Ultimately, shock waves may increase collagen synthesis by up-regulating IL-6 levels. IL-6 is a multifunctional inflammatory cytokine and a key regulator of connective tissue health that exhibits both pro- and anti-inflammatory effects and is released in response to mechanical loading. Although IL-6 may promote negative effects, there are many studies advocating a role for IL-6 in tendon adaptation. Importantly, IL-6 has been shown to stimulate fibroblasts to increase the production of various ECM components, including collagen. Studies have shown that the concentration of IL-6 can be significantly increased after ESWT. Collagen synthesis is thus increased (2).

### ESWT modulates inflammation in the tendon remodeling phase

The process of modulating inflammation is imperative to limit inflammation and initiate repair. The relief of the inflammatory response can encourage ECM remodeling and promote the resolution of tendinopathy. The use of non-steroidal anti-inflammatory drugs (NSAIDs) is often considered as a reliable pharmacological treatment among general conservative treatments. Though most people tolerate NSAIDs well, the elderly and certain patients with chronic diseases can experience side effects from NSAIDs that affect the cardiovascular function and cause gastric ulcers, among others, that are not seen with ESWT (65, 89). A number of clinical studies have shown that lower energy levels of ESWT have anti-inflammatory effects (65, 90, 91). The anti-inflammatory effect of ESWT in equine experiments was also demonstrated in a study by Silveira et al. They applied a low-energy ESWT of 0.11 mJ/mm2 to the superficial wound in the third metacarpal of equines and then found that the control group was 1.9 times more likely to have inflammation than the ESWT group. And the ESWT-treated group looked healthier than the control group (92).Yet, the mechanism of the anti-inflammatory effect of ESWT is not fully understood, but some reports suggest that NO plays a key role in this anti-inflammatory therapeutic effect. Physical factors such as shear forces can increase nitric oxide-derived NO levels in blood vessels for short periods of time, and low levels of shock waves may induce forces similar to laminar fluid shear stress. In the human body, there is a complex series of constitutively expressed nitric oxide synthases (CNOS) that ensure the ability to produce the appropriate amount of NO to regulate a number of biological reactions *in vivo*. This phenomenon is known as NO homeostasis. During inflammation, inducible NOS (iNOS), as another subtype of NOS, can produce a relatively large amount of NO in suitable time and space, which is beneficial for the body overall (93). Relatively large amounts of NO will lead to cytotoxic effects and may be able to swiftly clear invading organisms or damaged cells. NF-κB is a major nuclear factor that regulates the induction of inflammatory gene expression. NO would be a powerful inhibitor of NF-κB activation, if NO is at low physiological concentrations (estimated <50 nM) (94). When the level of NO is much lower than the physiological level, it may result in local activation of NF-κB because the inhibitory effect of NO on NF-κB has been lost (65).

There are many reports describing the non-enzymatic production of NO (95, 96). ESWT can induce non-enzymatic production of NO. The number of ESWT applications and the energy level applied can influence the amount of non-enzymatically produced NO. In addition, ESWT has been reported to rapidly increase the catalytic activity of eNOS in human umbilical vein endothelial cells (HUVEC). The different phosphorylation states of the enzyme balance the overall NO production. ESWT shifts the balance to the less tyrosine phosphorylated form and is also capable of promptly increasing the activity of eNOS. Of interest, ESWT may also allow eNOS to interact with other proteins, thereby triggering activation of eNOS activity, and ESWT may induce accumulation of NO in cells in the manner described above. Thus, the clinically observed anti-inflammatory effects of ESWT manifestation can be speculated to be a result of ESWT inducing a rapid upregulation of eNOS activity, sustained increase in NO output, and inhibition of NF-κB activation (65).

In addition, it has been shown that ESWT may regulate inflammation by stimulating Toll-Like Receptor 3 (TLR3), a part of the innate immune system involved in the recognition of double-stranded RNA (dsRNA) and fragmented deoxyribonucleic acid (DNA) from viruses (97, 98).TLR3 is also thus able to detect cytoplasmic RNA released by adjacent cells. Activation of TLR3 is associated with an early pro-inflammatory phase and a late anti-inflammatory response. It creates an environment for new angiogenesis and tissue repair in the injured tissue (99). Experimental studies revealed a complex interaction between two major cytokines, IL-6 and IL-10, following TLR3 stimulation. An intermediate phase showing inflammatory inhibition can be seen after the early pro-inflammatory initiation phase mediated by IL-6 and before the late anti-inflammatory limiting phase of IL-10. This regulation of the inflammatory response is a precondition for angiogenesis and repair in ischemic tissues. The current findings suggest that the tissue regenerative effects and modulating inflammatory effects of ESWT are at least partially mediated by TLR3 stimulation (100).

Substance P (SP) has been found to be strongly associated with a reduced inflammatory response in tendinopathies, and inflammatory symptoms are reduced in experiments with denervation or SP depletion (101).

SP and calcitonin gene-related peptide (CGRP) as neuropeptides released by primary sensory nerve terminals will cause the so-called neurogenic inflammation (102). It has been reported that after ESWT, degeneration of CGRP- and SP-positive nerve fibers was observed (49). This suggests that ESWT may suppress inflammation by reducing CGRP and SP. However, contrary to other studies, the results reported by Abed et al. found no histological changes in SP- and CGRP-containing nerve fibers in sheep that received ESWT (103). It suggests that we still need a large accumulation of evidence to prove whether ESWT can really suppress inflammation by affecting CGRP and SP-positive nerve fibers. On the other hand, tendinopathy can cause pain, and ESWT can relieve the pain of tendinopathy by depleting SP. It was shown that ESWT has analgesic effects similar to capsaicin and can cause SP depletion by causing SP release, which ultimately produces analgesic effects. This could explain the local pain and subsequent lasting pain relief that occurs after shock wave application to tendinopathy (45, 104).

**Figure 5:**
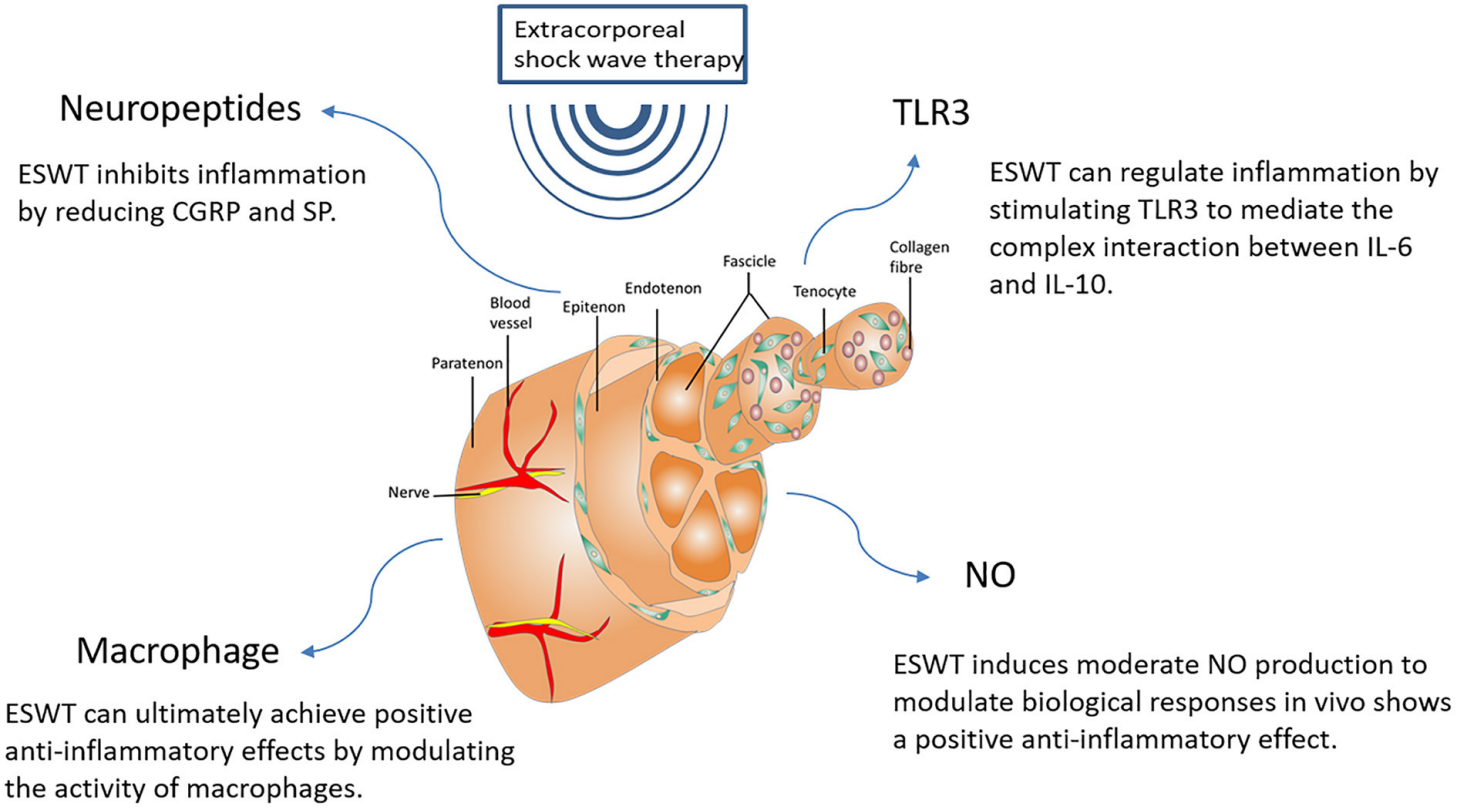
Major factors involved in the anti-inflammatory response of ESWT. NO, Nitric Oxide; CGRP, Calcitonin gene-related peptide; SP, Substance P; TLR3, Toll-Like Receptor 3; IL-6, Interleukin-6; IL-10, Interleukin-10.

Eventually, in addition to regulating NO, TLR3, and neuropeptides, ESWT can also affect cellular inflammation by downregulating leukocyte infiltration and regulating macrophage activity (55). Although shock waves do not induce activation of resting macrophages, they can suppress the inflammatory response by modulating macrophage activity. M1 macrophages are classical macrophages that produce pro-inflammatory cytokines and thus respond protectively to pathogens. M2 macrophages are heterogeneous macrophages with anti-inflammatory effects, but the mechanism of action is unclear. Low-energy ESWT can inhibit the pro-inflammatory characteristics of M1 macrophages and thus suppress the inflammatory response. At the same time, ESWT can promote the anti-inflammatory characteristics synergistically with M2 macrophages, so as to reap positive anti-inflammatory effects at last (105). ESWT may be used more often in the future for other different inflammatory diseases. We still need further studies to investigate the mechanisms by which ESWT regulates inflammation *in vivo*. Major factors involved in the anti-inflammatory response of ESWT are shown in Figure 5.

## Conclusion and perspectives

Tendinopathy has sophisticated pathophysiology. It consists of a transient acute inflammatory phase, but over a period of time, it gradually becomes a regressive disease. The management of tendinopathy is typically conservative. When other conservative treatments fail, in clinical practice, ESWT is commonly used as an efficient alternative to surgical management (12).

As a safe and widely used therapeutic tool, ESWT plays different roles in each of the three phases of tendon healing, eliciting different biological responses: during the inflammatory phase, ESWT increases the number of new blood vessels in the normal tendon-bone junction by regulating the release of growth factors and some other active substances, thus promoting an increase in nutrients and further accelerating tendon healing; while during the proliferative phase, ESWT stimulates tendon cell proliferation and collagen synthesis by generating mechanical stimulation; and in the remodeling phase, ESWT fosters ECM remodeling by regulating the tendon inflammatory response, which ultimately helps to achieve wound healing and tissue regeneration (2, 8, 106).

To avoid potential damage to tendon tissue from the shock wave, most of the time, low-energy ESWT with flux density <0.28 MJ/mm2 will be used clinically to cure tendon diseases (14). However, for animals, the tolerance to ESWT was different for different species. For example, rabbit Achilles tendon and rat Achilles tendon showed visible deleterious tissue effects at EFDs of 0.28 and 0.2 mJ/mm2, respectively (4, 57). Also, tendons in different locations of the same species have different tolerance levels. Maier et al. reported that the quadriceps tendon of rabbits was able to apply higher energy levels of ESWT than the rabbit Achilles tendon, but once it exceeded 0.5 mJ/mm2, the quadriceps tendon of rabbits also suffered damage (107). Though some relevant studies have given clear doses for application, most have been set empirically rather than through systematic and scientific studies. And the complications of ESWT were often reported as partial pain, residual pain, mild edema, and transient erythema at the site of ESWT application during treatment. These phenomena may be related to the lack of uniform treatment parameters for ESWT (108). The non-uniform treatment parameters also contribute to some extent to the unclear clinical efficacy of ESWT for tendinopathies. These studies remind us that more systematic and scientific studies are still needed in the coming years to formulate standard treatment parameters that can be broadly accepted for different species and for the same species but different tendon sites. This may solve many long-standing issues, such as improving the problem of hindlimbs having a higher recurrence rate than forelimbs after ESWT in equines. It will lead to better treatment results for animal tendon patients and help ESWT to be more widely used in the veterinary field.

It is also noteworthy that clinicians rarely make ESWT available as a single therapy, and future studies should evaluate more clinically oriented, individualized regimens in terms of clinical effectiveness. What's more, the inclusion of a placebo control group in the efficacy study of ESWT is necessary to prevent possible natural improvements in the early stages of tendinopathy from being mistaken for treatment effects and can maximize the reliability of the experiment. And due to the slow rate of metabolic turnover of the tendon tissue, studies should have a sufficiently long follow-up period to detect a treatment effect. These methodological issues could also explain some of the conflicting results of ESWT effectiveness. But there are still many limitations in the practical application: whether the owner is cooperative during the return visit and whether this will result in a low response rate; during the long follow-up period, are we able to ensure that the animal patients remain under controlled conditions; since ESWT can now be used by lay people, using ESWT without veterinary supervision may lead to misuse and abuse of ESWT in equine patients, etc.

A growing number of studies in recent years have started to apply ESWT in the treatment of patients with animal tendinopathies. The success of the treatment for the majority of animal patients with tendinopathy, such as horses, dogs and rabbits, shows the prospect of ESWT. Many studies have separately demonstrated the biological responses of ESWT in the different stages of tendinopathy repair, but there are no articles in the veterinary literature we searched that combine these biological responses with all three stages of tendinopathy repair simultaneously. In order to develop effective treatment strategies for tendinopathies, we must understand the biological response to all phases of the tendon healing event so that we can target and adapt treatment strategies when applying therapeutic modalities. Therefore, we systematically discuss and summarize the biological responses generated by ESWT in the three phases of tendinopathy repair and the mechanisms. In future studies, it is still necessary to further explore the biological response of ESWT in the treatment of tendinopathy in order to better improve the symptoms and prolong the therapeutic effect of tendon patients.

## Author contributions

YC, KL, and JL designed the present manuscript. YC drawn the manuscript. LJ, BZ, XuL, YL, XiL, LL, and XW performed a literature search and selected the studies to be performed. YC, HX, DW, and SL revised including the manuscript. All authors contributed to the article and approved the submitted version.

## Conflict of interest

The authors declare that the research was conducted in the absence of any commercial or financial relationships that could be construed as a potential conflict of interest.

## Publisher's note

All claims expressed in this article are solely those of the authors and do not necessarily represent those of their affiliated organizations, or those of the publisher, the editors and the reviewers. Any product that may be evaluated in this article, or claim that may be made by its manufacturer, is not guaranteed or endorsed by the publisher.

## References

[1] Wang JH. Mechanobiology of tendon. J Biomech. (2006) 39:1563–82. 10.1016/j.jbiomech.2005.05.011 16000201

[2] Waugh CM Morrissey D Jones E Riley GP Langberg H Screen HRC. In vivo biological response to extracorporeal shockwave therapy in human tendinopathy. Eur Cells Mat. (2015) 29:268–80. 10.22203/eCM.v029a20 25978115

[3] Del Buono A Battery L Denaro V Maccauro G Maffulli N. Tendinopathy and inflammation: some truths. Int J Immunopathol Pharmacol. (2011) 24(1 Suppl 2):45–50. 10.1177/03946320110241s209 21669137

[4] Bosch G Lin YL van Schie HT van De Lest CH Barneveld A van Weeren PR. Effect of extracorporeal shock wave therapy on the biochemical composition and metabolic activity of tenocytes in normal tendinous structures in ponies. Equine Vet J. (2007) 39:226–31. 10.2746/042516407x180408 17520973

[5] Nourissat G Berenbaum F Duprez D. Tendon injury: from biology to tendon repair. Nat Rev Rheumatol. (2015) 11:223–33. 10.1038/nrrheum.2015.26 25734975

[6] MacKay AV McOnie RC Riddell LP Robinson KA. Characterization of the use of shock wave therapy among equine veterinarians. Can Vet J. (2020) 61:990–3. 32879526 PMC7424940

[7] Chung B Wiley JP. Extracorporeal shockwave therapy. Sports Med. (2002) 32:851–65. 10.2165/00007256-200232130-00004 12392445

[8] d'Agostino MC Craig K Tibalt E Respizzi S. Shock wave as biological therapeutic tool: from mechanical stimulation to recovery and healing, through mechanotransduction. Int J Surgery. (2015) 24:147–53. 10.1016/j.ijsu.2015.11.030 26612525

[9] Modena DAO Soares CD Candido EC Chaim FDM Cazzo E Chaim EA. Effect of extracorporeal shock waves on inflammation and angiogenesis of integumentary tissue in obese individuals: stimulating repair and regeneration. Lasers Med Sci. (2021) 37:1289–297. 10.1007/s10103-021-03387-x 34365545

[10] Schnewlin M Lischer C. [Extracorporal shock wave therapy in veterinary medicine]. Schweizer Archiv Tierheilkunde. (2001) 143:227–32. 10.1590/S1516-35982001000400030 11407246

[11] Notarnicola A Moretti B. The biological effects of extracorporeal shock wave therapy (eswt) on tendon tissue. Muscles Ligam Tendons J. (2012) 2:33–7. 23738271 PMC3666498

[12] van der Worp H van den Akker-Scheek I van Schie H Zwerver J. ESWT for tendinopathy: technology and clinical implications. Knee Surgery Sports Traumatol Arthro. (2012) 21:1451–8. 10.1007/s00167-012-2009-3 22547246 PMC3657080

[13] Orhan Z Cam K Alper M Ozturan K. The effects of extracorporeal shock waves on the rat achilles tendon: is there a critical dose for tissue injury? Arch Orthopaedic Trauma Surg. (2003) 124:631–5. 10.1007/s00402-003-0598-5 14605827

[14] Wang C-J. Extracorporeal shockwave therapy in musculoskeletal disorders. J Orthopaedic Surg Res. (2012) 7:1–8. 10.1186/1749-799x-7-11 22433113 PMC3342893

[15] Mittermayr R Antonic V Hartinger J Kaufmann H Redl H Téot L. Extracorporeal shock wave therapy (ESWT) for wound healing: technology, mechanisms, clinical efficacy. Wound Rep Reg. (2012) 20:456–65. 10.1111/j.1524-475X.2012.00796.x 22642362

[16] Wang CJ. Extracorporeal shockwave therapy in musculoskeletal disorders. J Orthop Surg Res. (2012) 7:11. 10.1186/1749-799X-7-11 22433113 PMC3342893

[17] Alvarez L. Extracorporeal shockwave therapy for musculoskeletal pathologies. Vet Clin North Am Small Anim Pract. (2022) 52:1033–42. 10.1016/j.cvsm.2022.03.007 35715112

[18] Vetrano M d'Alessandro F Torrisi MR Ferretti A Vulpiani MC Visco V. Extracorporeal shock wave therapy promotes cell proliferation and collagen synthesis of primary cultured human tenocytes. Knee Surgery Sports Traumatol Arthro. (2011) 19:2159–68. 10.1007/s00167-011-1534-9 21617986

[19] Auersperg V Trieb K. Extracorporeal shock wave therapy: an update. EFORT Open Rev. (2020) 5:584–92. 10.1302/2058-5241.5.190067 33204500 PMC7608508

[20] Tognolo L Giordani F Biz C Bernini A Ruggieri P Stecco C. Myofascial points treatment with focused extracorporeal shock wave therapy (f-ESWT) for plantar fasciitis: an open label randomized clinical trial. Eur J Phys Rehabil Med. (2022) 58:85–93. 10.23736/s1973-9087.21.06814-3 34786906 PMC9980534

[21] Fan Y Feng Z Cao J Fu W. Efficacy of extracorporeal shock wave therapy for achilles tendinopathy: a meta-analysis. Orthop J Sports Med. (2020) 8:2325967120903430. 10.1177/2325967120903430 33283015 PMC7686643

[22] Rompe JD Schoellner C Nafe B. Evaluation of low-energy extracorporeal shock-wave application for treatment of chronic plantar fasciitis. J Bone Joint Surg Am. (2002) 84:335–41. 10.2106/00004623-200203000-00001 11886900

[23] Lai TW Ma HL Lee MS Chen PM Ku MC. Ultrasonography and clinical outcome comparison of extracorporeal shock wave therapy and corticosteroid injections for chronic plantar fasciitis: a randomized controlled trial. J Musculoskelet Neuronal Interact. (2018) 18:47–54. 29504578 PMC5881128

[24] Çaglar Okur S Aydin A. Comparison of extracorporeal shock wave therapy with custom foot orthotics in plantar fasciitis treatment: a prospective randomized one-year follow-up study. J Musculoskelet Neuronal Interact. (2019) 19:178–86. 31186388 PMC6587088

[25] Gleitz M Hornig K. [Trigger points - diagnosis and treatment concepts with special reference to extracorporeal shockwaves]. Orthopade. (2012) 41:113–25. 10.1007/s00132-011-1860-0 22349369

[26] Ramon S Gleitz M Hernandez L Romero LD. Update on the efficacy of extracorporeal shockwave treatment for myofascial pain syndrome and fibromyalgia. Int J Surg. (2015) 24:201–6. 10.1016/j.ijsu.2015.08.083 26363497

[27] Giordani F Bernini A Müller-Ehrenberg H Stecco C Masiero S. A global approach for plantar fasciitis with extracorporeal shockwaves treatment. Eur J Transl Myol. (2019) 29:8372. 10.4081/ejtm.2019.8372 31579484 PMC6767838

[28] Melese H Alamer A Getie K Nigussie F Ayhualem S. Extracorporeal shock wave therapy on pain and foot functions in subjects with chronic plantar fasciitis: systematic review of randomized controlled trials. Disabil Rehabil. (2021) 16:1–8. 10.1080/09638288.2021.1928775 34038642

[29] Buchbinder R Ptasznik R Gordon J Buchanan J Prabaharan V Forbes A. Ultrasound-guided extracorporeal shock wave therapy for plantar fasciitis: a randomized controlled trial. JAMA. (2002) 288:1364–72. 10.1001/jama.288.11.1364 12234230

[30] Speed CA Nichols D Wies J Humphreys H Richards C Burnet S. Extracorporeal shock wave therapy for plantar fasciitis. A double blind randomised controlled trial. J Orthop Res. (2003) 21:937–40. 10.1016/s0736-0266(03)00048-2 12919884

[31] Greve JMDA Grecco MV Santos-Silva PR. Comparison of radial shockwaves and conventional physiotherapy for treating plantar fasciitis. Clinics. (2009) 64:97–103. 10.1590/s1807-59322009000200006 19219314 PMC2666476

[32] Notarnicola A Moretti B. The biological effects of extracorporeal shock wave therapy (eswt) on tendon tissue. Muscles Ligaments Tendons J. (2012) 2:33–7. 23738271 PMC3666498

[33] Rompe JD Decking J Schoellner C Theis C. Repetitive low-energy shock wave treatment for chronic lateral epicondylitis in tennis players. Am J Sports Med. (2004) 32:734–43. 10.1177/0363546503261697 15090392

[34] Haake M König IR Decker T Riedel C Buch M Müller HH. Extracorporeal shock wave therapy in the treatment of lateral epicondylitis : a randomized multicenter trial. J Bone Joint Surg Am. (2002) 84:1982–91. 10.2106/00004623-200211000-00012 12429759

[35] Crowther MAA Bannister GC Huma H Rooker GD. A prospective, randomised study to compare extracorporeal shock-wave therapy and injection of steroid for the treatment of tennis elbow. J Bone Joint Surg. (2002) 84-B:678–9. 10.1302/0301-620x.84b5.0840678 12188483

[36] Gerdesmeyer L Wagenpfeil S Haake M Maier M Loew M Wörtler K. Extracorporeal shock wave therapy for the treatment of chronic calcifying tendonitis of the rotator cuff. JAMA. (2003) 290:2573–580. 10.1001/jama.290.19.2573 14625334

[37] Cacchio A Paoloni M Barile A Don R de Paulis F Calvisi V. Effectiveness of radial shock-wave therapy for calcific tendinitis of the shoulder: single-blind, randomized clinical study. Phys Ther. (2006) 86:672–82. 10.1093/ptj/86.5.672 16649891

[38] Sabeti-Aschraf M Dorotka R Goll A Trieb K. Extracorporeal shock wave therapy in the treatment of calcific tendinitis of the rotator cuff. Am J Sports Med. (2005) 33:1365–8. 10.1177/0363546504273052 16002492

[39] van Rijn D van den Akker-Scheek I Steunebrink M Diercks RL Zwerver J van der Worp H. Comparison of the effect of 5 different treatment options for managing patellar tendinopathy: a secondary analysis. Clin J Sport Med. (2019) 29:181–7. 10.1097/JSM.0000000000000520 31033610

[40] van der Worp H Zwerver J Hamstra M van den Akker-Scheek I Diercks RL. No difference in effectiveness between focused and radial shockwave therapy for treating patellar tendinopathy: a randomized controlled trial. Knee Surg Sports Traumatol Arthrosc. (2014) 22:2026–32. 10.1007/s00167-013-2522-z 23666379

[41] Zwerver J Hartgens F Verhagen E van der Worp H van den Akker-Scheek I Diercks RL. No effect of extracorporeal shockwave therapy on patellar tendinopathy in jumping athletes during the competitive season: a randomized clinical trial. Am J Sports Med. (2011) 39:1191–9. 10.1177/0363546510395492 21285447

[42] Furia JP Rompe JD Cacchio A Del Buono A Maffulli N. A single application of low-energy radial extracorporeal shock wave therapy is effective for the management of chronic patellar tendinopathy. Knee Surg Sports Traumatol Arthrosc. (2013) 21:346–50. 10.1007/s00167-012-2057-8 22627667

[43] Korakakis V Whiteley R Tzavara A Malliaropoulos N. The effectiveness of extracorporeal shockwave therapy in common lower limb conditions: a systematic review including quantification of patient-rated pain reduction. Br J Sports Med. (2018) 52:387–407. 10.1136/bjsports-2016-097347 28954794

[44] Seil R Wilmes P Nuhrenborger C. Extracorporeal shock wave therapy for tendinopathies. Expert Rev Med Devices. (2006) 3:463–70. 10.1586/17434440.3.4.463 16866643

[45] Ohtori S Inoue G Mannoji C Saisu T Takahashi K Mitsuhashi S. Shock wave application to rat skin induces degeneration and reinnervation of sensory nerve fibres. Neurosci Lett. (2001) 315:57–60. 10.1016/s0304-3940(01)02320-5 11711214

[46] Dahlberg JA McClure SR Evans RB Reinertson EL. Force platform evaluation of lameness severity following extracorporeal shock wave therapy in horses with unilateral forelimb lameness. J Am Vet Med Assoc. (2006) 229:100–3. 10.2460/javma.229.1.100 16817722

[47] Imboden I Waldern NM Wiestner T Lischer CJ Ueltschi G Weishaupt MA. Short term analgesic effect of extracorporeal shock wave therapy in horses with proximal palmar metacarpal/plantar metatarsal pain. Vet J. (2009) 179:50–9. 10.1016/j.tvjl.2007.09.020 18069025

[48] Lian O Dahl J Ackermann PW Frihagen F Engebretsen L Bahr R. Pronociceptive and antinociceptive neuromediators in patellar tendinopathy. Am J Sports Med. (2006) 34:1801–8. 10.1177/0363546506289169 16816149

[49] Hausdorf J Lemmens MA Heck KD Grolms N Korr H Kertschanska S. Selective loss of unmyelinated nerve fibers after extracorporeal shockwave application to the musculoskeletal system. Neuroscience. (2008) 155:138–44. 10.1016/j.neuroscience.2008.03.062 18579315

[50] Carulli C Tonelli F Innocenti M Gambardella B Muncibi F Innocenti M. Effectiveness of extracorporeal shockwave therapy in three major tendon diseases. J Orthop Traumatol. (2016) 17:15–20. 10.1007/s10195-015-0361-z 26135551 PMC4805637

[51] Melzack R. Sensory modulation of pain. Int Rehabil Med. (1979) 1:111–5. 10.3109/03790797909163937 233309

[52] Rompe JD Küllmer K Vogel J Eckardt A Wahlmann U Eysel P. Extrakorporale Stoßwellentherapie. Der Orthopäde. (1997) 26:215–28. 10.1007/pl00003377 9198795

[53] Arvind V Huang AH. Reparative and maladaptive inflammation in tendon healing. Front Bioengin Biotechnol. (2021) 09:1–16. 10.3389/fbioe.2021.719047 34350166 PMC8327090

[54] Waugh CM Morrissey D Jones E Riley GP Langberg H Screen HR. *In vivo* biological response to extracorporeal shockwave therapy in human tendinopathy. Eur Cell Mater. (2015) 29:268–280; discussion 280. 10.22203/ecm.v029a20 25978115

[55] Heimes D Wiesmann N Eckrich J Brieger J Mattyasovszky S Proff P. *In vivo* modulation of angiogenesis and immune response on a collagen matrix via extracorporeal shockwaves. Int J Mol Sci. (2020) 21:1–26. 10.3390/ijms21207574 33066403 PMC7589066

[56] Millar NL Murrell GAC McInnes IB. Inflammatory mechanisms in tendinopathy – towards translation. Nat Rev Rheumatol. (2017) 13:110–22. 10.1038/nrrheum.2016.213 28119539

[57] Bosch G Mos M Binsbergen R Schie HTM Lest CHA Weeren PR. The effect of focused extracorporeal shock wave therapy on collagen matrix and gene expression in normal tendons and ligaments. Equine Vet J. (2009) 41:335–41. 10.2746/042516409x370766 19562893

[58] Aschermann I Noor S Venturelli S Sinnberg T Mnich CD Busch C. Extracorporal shock waves activate migration, proliferation and inflammatory pathways in fibroblasts and keratinocytes, and improve wound healing in an open-label, single-arm study in patients with therapy-refractory chronic leg ulcers. Cell Physiol Biochem. (2017) 41:890–906. 10.1159/000460503 28222435

[59] Chen YJ Wang CJ Yang KD Kuo YR Huang HC Huang YT. Extracorporeal shock waves promote healing of collagenase-induced achilles tendinitis and increase TGF-beta1 and IGF-I expression. J Orthop Res. (2004) 22:854–61. 10.1016/j.orthres.2003.10.013 15183445

[60] Uzun C Erdal N Gürgül S Kalayc,i D Yilmaz SN. Özdemir AA, et al. Comparison of the effects of pulsed electromagnetic field and extracorporeal shockwave therapy in a rabbit model of experimentally induced achilles tendon injury. Bioelectromagnetics. (2020) 42:128–45. 10.1002/bem.22314 33368423

[61] Orhan Z Ozturan K Guven A Cam K. The effect of extracorporeal shock waves on a rat model of injury to tendo achillis. J Bone Joint Surg. (2004) 86-B, 613–8. 10.1302/0301-620x.86b4.14692 15174564

[62] Wang C-J Huang H-Y Pai C-H. Shock wave-enhanced neovascularization at the tendon-bone junction: an experiment in dogs. J Foot Ankle Surgery. (2002) 41:16–22. 11858601 10.1016/s1067-2516(02)80005-9

[63] Hatanaka K Ito K Shindo T Kagaya Y Ogata T Eguchi K. Molecular mechanisms of the angiogenic effects of low-energy shock wave therapy: roles of mechanotransduction. Am J Physiol Cell Physiol. (2016) 311:C378–85. 10.1152/ajpcell.00152.2016 27413171

[64] Wang C-J Wang F-S Yang KD Wen L-H. Hsu C-C Huang C-S. Shock wave therapy induces neovascularization at the tendon–bone junction. A study in rabbits. J Orthop Res. (2003) 21:984–9. 10.1016/s0736-0266(03)00104-9 14554209

[65] Mariotto S de Prati A Cavalieri E Amelio E Marlinghaus E Suzuki H. Extracorporeal shock wave therapy in inflammatory diseases: molecular mechanism that triggers anti-inflammatory action. Curr Med Chem. (2009) 16:2366–72. 10.2174/092986709788682119 19601786

[66] Weihs AM Fuchs C Teuschl AH Hartinger J Slezak P Mittermayr R. Shock wave treatment enhances cell proliferation and improves wound healing by ATP release-coupled extracellular signal-regulated kinase (ERK) activation. J Biol Chem. (2014) 289:27090–104. 10.1074/jbc.M114.580936 25118288 PMC4175346

[67] Chao Y-H Tsuang Y-H Sun J-S Chen L-T Chiang Y-F Wang C-C. Effects of shock waves on tenocyte proliferation and extracellular matrix metabolism. Ultrasound Med Biol. (2008) 34:841–52. 10.1016/j.ultrasmedbio.2007.11.002 18222032

[68] Ito K Fukumoto Y Shimokawa H. Extracorporeal shock wave therapy as a new and non-invasive angiogenic strategy. Tohoku J Exp Med. (2009) 219:1–9. 10.1620/tjem.219.1 19713678

[69] Goertz O Lauer H Hirsch T Ring A Lehnhardt M Langer S. Extracorporeal shock waves improve angiogenesis after full thickness burn. Burns. (2012) 38:1010–8. 10.1016/j.burns.2012.02.018 22445836

[70] Kersh KD McClure SR Van Sickle D Evans RB. The evaluation of extracorporeal shock wave therapy on collagenase induced superficial digital flexor tendonitis. Vet Comp Orthop Traumatol. (2006) 19:99–105. 16810352

[71] Lee J-W Bae S-H Jeong J-W Kim SH Kim K-W. Hypoxia-inducible factor (HIF-1)α: its protein stability and biological functions. Exp Mol Med. (2004) 36:1–12. 10.1038/emm.2004.1 15031665

[72] Tepeköylü C Wang F.-S. Kozaryn R Albrecht-Schgoer K Theurl M. Shock wave treatment induces angiogenesis and mobilizes endogenous CD31/CD34-positive endothelial cells in a hindlimb ischemia model: implications for angiogenesis and vasculogenesis. J Thor Cardiov Surg. (2013) 146:971–8. 10.1016/j.jtcvs.2013.01.017 23395097

[73] Ha CH Kim S Chung J An SH Kwon K. Extracorporeal shock wave stimulates expression of the angiogenic genes via mechanosensory complex in endothelial cells: Mimetic effect of fluid shear stress in endothelial cells. Int J Cardiol. (2013) 168:4168–77. 10.1016/j.ijcard.2013.07.112 23915523

[74] Tsai W-C Hsu C-C Tang F-T Chou S-W Chen Y-J Pang J-HS. Ultrasound stimulation of tendon cell proliferation and upregulation of proliferating cell nuclear antigen. J Ortho Res. (2005) 23:970–6. 10.1016/j.orthres.2004.11.013 16023014

[75] Wang L Kong W Liu B Zhang X. Proliferating cell nuclear antigen promotes cell proliferation and tumorigenesis by up-regulating STAT3 in non-small cell lung cancer. Biomed Pharmacother. (2018) 104:595–602. 10.1016/j.biopha.2018.05.071 29803172

[76] Stoll C John T Endres M Rosen C Kaps C Kohl B. Extracellular matrix expression of human tenocytes in three-dimensional air-liquid and PLGA cultures compared with tendon tissue: implications for tendon tissue engineering. J Orthop Res. (2010) 28:1170–7. 10.1002/jor.21109 20187116

[77] Orhan Z Alper M Akman Y Yavuz O Yalçiner A. An experimental study on the application of extracorporeal shock waves in the treatment of tendon injuries: preliminary report. J Orthop Sci. (2001) 6:566–70. 10.1007/s007760100013 11793180

[78] Hsu RW Hsu WH Tai CL Lee KF. Effect of shock-wave therapy on patellar tendinopathy in a rabbit model. J Orthop Res. (2004) 22:221–7. 10.1016/s0736-0266(03)00138-4 14656684

[79] Chang J Most D Stelnicki E Siebert JW Longaker MT Hui K. Gene expression of transforming growth factor beta-1 in rabbit zone II flexor tendon wound healing: evidence for dual mechanisms of repair. Plast Reconst Surgery. (1997) 100:937–44. 10.1097/00006534-199709001-00016 9290662

[80] Lijnen PJ Petrov VV Fagard RH. Induction of cardiac fibrosis by transforming growth factor-beta(1). Mol Genet Metab. (2000) 71:418–35. 10.1006/mgme.2000.3032 11001836

[81] Fujita T Shiba H Sakata M Uchida Y Ogawa T Kurihara H. Effects of transforming growth factor-beta 1 and fibronectin on SPARC expression in cultures of human periodontal ligament cells. Cell Biol Int. (2002) 26:1065–72. 10.1006/cbir.2002.0966 12468382

[82] Yang G Crawford RC Wang JHC. Proliferation and collagen production of human patellar tendon fibroblasts in response to cyclic uniaxial stretching in serum-free conditions. J Biomech. (2004) 37:1543–50. 10.1016/j.jbiomech.2004.01.005 15336929

[83] Vodovotz Y Chesler L Chong H Kim SJ Simpson JT DeGraff W. Regulation of transforming growth factor beta1 by nitric oxide. Can Res. (1999) 59:2142–9. 10232601

[84] Chang J Most D Thunder R Mehrara B Longaker MT Lineaweaver WC. Molecular studies in flexor tendon wound healing: the role of basic fibroblast growth factor gene expression. J Hand Surgery. (1998) 23:1052–8. 10.1016/s0363-5023(98)80015-4 9848558

[85] Takahashi S Fujita T Yamamoto A. Role of nuclear factor-κB in gastric ulcer healing in rats. Am J Physiol Gastroint Liver Physiol. (2001) 280:G1296–304. 10.1152/ajpgi.2001.280.6.G1296 11352824

[86] Motani A Forster L Tull S ÄNggÅRd EE Ferns GAA. Insulin-like growth factor-I modulates monocyte adhesion to EAhy 926 endothelial cells. Int J Exp Pathol. (2003) 77:31–5. 10.1046/j.1365-2613.1996.960098.x 8664144 PMC2691619

[87] Tang JB Xu Y Ding F Wang XT. Tendon healing in vitro: promotion of collagen gene expression by bFGF with NF-κB gene activation. J Hand Surgery. (2003) 28:215–20. 10.1053/jhsu.2003.50052 12671851

[88] Rosso F Bonasia DE Marmotti A Cottino U Rossi R. Mechanical stimulation (pulsed electromagnetic fields “PEMF” and extracorporeal shock wave therapy “ESWT”) and tendon regeneration: a possible alternative. Front Aging Neurosci. (2015) 7:1052–1058. 10.3389/fnagi.2015.00211 26617513 PMC4637423

[89] Dakin SG Dudhia J Smith RK. Resolving an inflammatory concept: the importance of inflammation and resolution in tendinopathy. Vet Immunol Immunopathol. (2014) 158:121–7. 10.1016/j.vetimm.2014.01.007 24556326 PMC3991845

[90] Kuo YR Wu WS Hsieh YL Wang FS Wang CT Chiang YC. Extracorporeal shock wave enhanced extended skin flap tissue survival via increase of topical blood perfusion and associated with suppression of tissue pro-inflammation. J Surg Res. (2007) 143:385–92. 10.1016/j.jss.2006.12.552 17720194

[91] Davis TA Stojadinovic A Anam K Amare M Naik S Peoples GE. Extracorporeal shock wave therapy suppresses the early proinflammatory immune response to a severe cutaneous burn injury. Int Wound J. (2009) 6:11–21. 10.1111/j.1742-481X.2008.00540.x 19291111 PMC7951765

[92] Silveira A Koenig JB Arroyo LG Trout D Moens NM LaMarre J. Effects of unfocused extracorporeal shock wave therapy on healing of wounds of the distal portion of the forelimb in horses. Am J Vet Res. (2010) 71:229–34. 10.2460/ajvr.71.2.229 20113232

[93] Rastaldo R Pagliaro P Cappello S Penna C Mancardi D Westerhof N. Nitric oxide and cardiac function. Life Sci. (2007) 81:779–93. 10.1016/j.lfs.2007.07.019 17707439

[94] Colasanti M Persichini T. Nitric oxide: an inhibitor of NF-κB/Rel system in glial cells. Brain Res Bull. (2000) 52:155–61. 10.1016/s0361-9230(00)00262-8 10822156

[95] Moroz LL Norby SW Cruz L Sweedler JV Gillette R Clarkson RB. Non-enzymatic production of nitric oxide (NO) from NO synthase inhibitors. Biochem Biophys Res Commun. (1998) 253:571–6. 10.1006/bbrc.1998.9810 9918769

[96] Oldreive C Rice-Evans C. The mechanisms for nitration and nitrotyrosine formationin vitroandin vivo: impact of diet. Free Rad Res. (2009) 35:215–31. 10.1080/10715760100300761 11697121

[97] Alexopoulou L Holt AC Medzhitov R Flavell RA. Recognition of double-stranded RNA and activation of NF-κB by Toll-like receptor 3. Nature. (2001) 413:732–8. 10.1038/35099560 11607032

[98] Takeda K Kaisho T Akira S. Toll-like receptors. Ann Rev Immunol. (2003) 21:335–76. 10.1146/annurev.immunol.21.120601.141126 12524386

[99] Chi H Barry SP Roth RJ Wu JJ Jones EA Bennett AM. Dynamic regulation of pro- and anti-inflammatory cytokines by MAPK phosphatase 1 (MKP-1) in innate immune responses. Proc Natl Acad Sci USA. (2006) 103:2274–9. 10.1073/pnas.0510965103 16461893 PMC1413743

[100] Holfeld J Tepeköylü C Kozaryn R Urbschat A Zacharowski K Grimm M. Shockwave therapy differentially stimulates endothelial cells: implications on the control of inflammation via toll-like receptor 3. Inflammation. (2013) 37:65–70. 10.1007/s10753-013-9712-1 23948864

[101] Garrett NE Cruwys SC L Kidd B Tomlinson DR. Effect of capsaicin on substance P and nerve growth factor in adjuvant arthritic rats. Neurosci Lett. (1997) 230:5–8. 10.1016/s0304-3940(97)00458-8 9259450

[102] Richardson JD Vasko MR. Cellular mechanisms of neurogenic inflammation. J Pharmacol Exp Ther. (2002) 302:839–45. 10.1124/jpet.102.032797 12183638

[103] Abed JM McClure SR Yaeger MJ Evans RB. Immunohistochemical evaluation of substance P and calcitonin gene-related peptide in skin and periosteum after extracorporeal shock wave therapy and radial pressure wave therapy in sheep. Am J Vet Res. (2007) 68:323–8. 10.2460/ajvr.68.3.323 17331023

[104] Maier M Averbeck B Milz S Refior HJ Schmitz C. Substance P and prostaglandin E2 release after shock wave application to the rabbit femur. Clin Orthop Relat Res. (2003) 302:237–45. 10.1097/01.blo.0000030173.56585.8f 12579024

[105] Sukubo NG Tibalt E Respizzi S Locati M d'Agostino MC. Effect of shock waves on macrophages: a possible role in tissue regeneration and remodeling. Int J Surgery. (2015) 24:124–30. 10.1016/j.ijsu.2015.07.719 26291028

[106] Liao CD Xie GM Tsauo JY Chen HC Liou TH. Efficacy of extracorporeal shock wave therapy for knee tendinopathies and other soft tissue disorders: a meta-analysis of randomized controlled trials. BMC Muscul Disord. (2018) 19:278. 10.1186/s12891-018-2204-6 30068324 PMC6090995

[107] Maier M Tischer T Milz S Weiler C Nerlich A Pellengahr C. Dose-related effects of extracorporeal shock waves on rabbit quadriceps tendon integrity. Arch Orthop Trauma Surg. (2002) 122:436–41. 10.1007/s00402-002-0420-9 12442179

[108] Al-Abbad H Simon JV. The effectiveness of extracorporeal shock wave therapy on chronic achilles tendinopathy. Foot Ankle Int. (2013) 34:33–41. 10.1177/1071100712464354 23386759

